# Certification of breast centres in Germany: proof of concept for a prototypical example of quality assurance in multidisciplinary cancer care

**DOI:** 10.1186/1471-2407-9-228

**Published:** 2009-07-14

**Authors:** Sara Y Brucker, Michael Bamberg, Walter Jonat, Matthias W Beckmann, Andreas Kämmerle, Rolf Kreienberg, Diethelm Wallwiener

**Affiliations:** 1 German Society of Senology, and Department of Obstetrics and Gynaecology, University of Tübingen, Tübingen, Germany; 2 German Cancer Society and Department of Radiooncology, University of Tübingen, Tübingen, Germany; 3 German Society for Gynaecology and Obstetrics and Department of Obstetrics and Gynaecology, Christian-Albrechts-Universität, Kiel, Germany; 4 Working Group on Gynaecological Oncology of the German Society for Gynaecology and Obstetrics, Munich, Germany; 5 Department of Obstetrics and Gynaecology, University of Erlangen, Erlangen, Germany; 6 OnkoZert GmbH, Ulm, Germany; 7 Certification Committee of the German Cancer Society and German Society of Senology, Berlin, Germany; 8 Department of Obstetrics and Gynaecology of the University of Ulm, Ulm, Germany

## Abstract

**Background:**

The main study objectives were: to develop a set of requirements of comprehensive breast centres; to establish a nationwide voluntary certification programme for breast centres based on such requirements, a certified quality management system (QMS), and scheduled independent, external audits and periodic recertification; and to demonstrate the general acceptance of such a certification programme with a view to introducing similar certification programmes for other major cancers.

**Methods:**

Breast centres introduced a QMS and voluntarily participated in an external certification procedure based on guideline-derived Requirements of Breast Centres specifically developed for the application procedure, all subsequent audits and recertification. All data (numbers of pending and successful applications, sites/centre, etc.) were collected by a newly founded, independent organisation for certification of cancer services delivery. Data analysis was descriptive.

**Results:**

Requirements of Breast Centres were developed by the German Cancer Society (DKG), the German Society of Senology (DGS) and other relevant specialist medical societies in the form of a questionnaire comprising 185 essential items based on evidence-based guidelines and the European Society of Breast Cancer Specialists' (EUSOMA) requirements of specialist breast units. From late 2002 to mid 2008, the number of participating breast centres rose from 1 to 175. As of mid 2008, 77% of an estimated 50,000 new breast cancers in Germany were diagnosed and treated at certified breast centres, 78% of which were single-site centres.

**Conclusion:**

Nationwide voluntary certification of breast centres is feasible and well accepted in Germany. Dual certification of breast centres that involves certification of breast services to guideline-derived requirements in conjunction with independent certification of a mandatory QMS can serve as a model for other multidisciplinary site-specific cancer centres.

## Background

Breast cancer, like many other malignancies, is regarded today as a systemic disease requiring multimodal, multidisciplinary treatment to achieve the best possible outcomes [1,2]. In recent years, the provision of evidence-based cancer care and the implementation and maintenance of quality assurance procedures have moved into the centre of attention as two key areas of modern multidisciplinary oncology [3].

In this context, the quest for strategies to reduce breast cancer morbidity and mortality has focused on the setting required for the optimal treatment of breast cancer because epidemiological and clinical data have pointed to the urgent need for the reorganisation and improved delivery of breast cancer care [4,5].

In its 2003 and 2006 resolutions on breast cancer in the European Union (EU) and the enlarged EU, respectively, the European Parliament (EP) called on the EU member states to create, by 2008, the conditions required for a 25% reduction in average breast cancer mortality and for reducing to 5% the disparity between EU countries in 5-year survival [6,7]. Both resolutions also called for the establishment of a network of certified multidisciplinary breast centres which essentially meet the core criteria set by the European Society of Breast Cancer Specialists (EUSOMA) in 2000 and 2004 in their Requirements of a specialist Breast Unit [8,9]. These in turn were based on, inter alia, the key aspects of specialisation and centralisation of cancer treatment as recommended by the 1995 Calman-Hines report, which proposed a policy framework for commissioning cancer services in the United Kingdom [10].

In view of the high incidence of breast cancer and the recognised necessity of providing appropriate multidisciplinary treatment, the management of this cancer can be considered as a prototypical example of a complete process chain ranging from early detection and diagnosis to treatment and follow-up. Under this aspect, breast cancer care requires elements of quality management, particularly at the various interfaces along the multidisciplinary and bisectoral, i.e. in- and out-patient, process chain [4,5]. Moreover, all health care providers in Germany are required by national law to perform quality assurance, although certification or other external monitoring of a complete quality management system (QMS) is not mandatory [11].

Against this backdrop, and in line with the European policies to reduce breast cancer mortality, which date back to the early 1990s, the German Cancer Society (*Deutsche Krebsgesellschaft*, DKG), the multidisciplinary German Society of Senology (*Deutsche Gesellschaft für Senologie*, DGS) and other relevant German specialist medical societies, including the German Society for Gynaecology and Obstetrics (*Deutsche Gesellschaft für Gynäkologie und Geburtshilfe*, DGGG), undertook to develop and implement a nationwide dual certification programme for breast centres. Certification means the assessment of an organisation by an accredited third party to show that the applicant abides by principles set out in a specific standard to ensure best practice, conformity with the standard being attested by a certificate. The planned certification programme was to involve certification of breast cancer services on the one hand and certification of a mandatory QMS on the other [12,11]. In the absence of specific legal requirements, certification was to be voluntary. Certification of service delivery to specific Requirements of Breast Centres (*Fachliche Anforderungen an Brustzentren*, FAB) [13] was to be organised and conducted on behalf of the DKG and the relevant specialist medical societies by a newly established, independent organisation for services related to the certification of cancer centres. Specifically, the FAB were to be set by the DKG and DGS mainly on the basis of the two multidisciplinary, evidence-based national level-3 guidelines [14-16] and the EUSOMA Requirements [8,9].

Based on the data collected from late 2002 to mid 2008 we aim to show in the following feasibility and proof-of-concept study that it has proved possible in Germany to establish a well-accepted, voluntary dual certification programme for breast centres that involves both certification to the purpose-developed DKG/DGS Requirements of Breast Centres (FAB) by a newly founded, independent organisation for certification of cancer services delivery, and certification of a mandatory QMS to ISO 9001 or a similar standard (KTQ) by German third-party certifying bodies. We also aim to show that certification has resulted in the centralisation of breast cancer services and that the great majority of breast cancer patients today are diagnosed and treated at specialist breast centres in Germany. Moreover, to quantify the quality of breast cancer services and improvements in the quality of breast cancer care, a Germany-wide voluntary benchmarking programme was established in parallel with the certification programme. Benchmarking is another key instrument for quality assurance and enhancement, and the first results from the initial 2003–2007 period of the benchmarking programme have recently been reported by Brucker and associates [17].

## Methods

### Study rationale, design and objectives

The present study was conducted under the auspices of the German Cancer Society (*Deutsche Krebsgesellschaft*; DKG) and the German Society of Senology (*Deutsche Gesellschaft für Senologie*; DGS) as a prospective, iterative interventional multicentre feasibility study. The study was prospective in that it was conducted to develop a detailed set of definitive Requirements of Breast Centres based on the Requirements of a Specialist Breast Unit set by the European Society of Breast Cancer Specialist (EUSOMA) [8,9], and two multidisciplinary, evidence-based national level-3 guidelines [16,15,14], and to create and implement a voluntary certification programme for breast centres in Germany [11]. The study was interventional in two respects. Firstly, a collaborative network of breast centres needed to be established to enable external collection of data. Secondly, external data collection and breast centre certification required the establishment of a provider of certification services for the delivery of specialist (breast) cancer services that was separate from, and independent of, the participating clinical institutions. This necessitated the foundation of an organisation that would be commissioned to conduct programmes for the certification of site-specific cancer centres on behalf of the DKG and DGS.

The **rationale **for the present study [4,5] was based on the following cornerstones:

1. specialisation (a high annual hospital volume of > 150 newly diagnosed breast cancers [18] and a caseload of ≥ 50 primary operations/year per breast surgeon [8]);

2. multidisciplinarity [19-22];

3. QMS certification by a third-party certifying body to an internationally recognised standard such as ISO 9001; and

4. benchmarking of the quality of breast cancer care [17].

The study followed a **design **with four, partly overlapping phases to provide proof of concept for the feasibility and acceptance of a nationwide voluntary certification programme for breast centres in Germany.

**Phase I:** A set of specific, largely guideline-based Requirements of Breast Centres (*Fachliche Anforderungen an Brustzentren*; FAB) was to be developed as the basis for the certification of multidisciplinary cancer services delivery.

**Phase II:** Pilot certification to the FAB was to be performed for a small number of breast centres in addition to separate mandatory third-party certification of a previously implemented hospital quality management system (QMS).

**Phase III:** Recertification of the pilot breast centres was to demonstrate proof of concept for the DKG/DGS programme for dual certification of breast centres.

**Phase IV:** Certification and recertification of other breast centres in Germany was to extend the proof of concept to the multicentre level.

The **objectives **of the present prospective iterative study were:

• to develop a set of Requirements of Breast Centres, primarily for Germany, under the joint direction of the German Cancer Society (DKG) and the German Society of Senology (DGS) in collaboration with other relevant German specialist medical societies;

• to establish an independent organisation that would co-ordinate certification to the DKG/DGS Requirements (FAB) on behalf of the DKG and the DGS;

• to create an appropriate procedure for DKG/DGS certification of breast centres having a mandatory, third-party-certified QMS;

• to build a nationwide voluntary network that would ultimately encompass all breast centres in Germany and, later, other countries; and

• to demonstrate the feasibility and general acceptance of the programme, thus providing a proof of concept for the DGK/DGS certification programme.

The final objective of the study was to show that a nationwide certification programme for breast centres would be associated with much improved guideline compliance and centralisation of breast services during the period from late 2002 to mid 2008.

### Participating institutions

Specialist breast centres and hospitals with breast care units focussing on breast cancer care in Germany, Austria (as of 2003), Switzerland and the partly German-speaking regions of northern Italy (both as of 2006) were invited to participate on a voluntary basis in an independent, external, scientifically based certification programme jointly developed by the German Cancer Society (DKG) and the German Society of Senology (DGS) and in collaboration with independent certifying bodies (see below).

### DKG/DGS Requirements of Breast Centres (FAB)

Starting from an early checklist of basic requirements in 2000, a detailed set of requirements was to be developed by the DKG, DGS and other relevant specialist medical societies. These DKG/DGS Requirements of Breast Centres (*Fachliche Anforderungen an Brustzentren*; FAB) were to include the EUSOMA Requirements [8,9] as well as items derived from the two relevant multidisciplinary, evidence-based national level-3 guidelines [15,16] and additional items based on German legal requirements.

### Quality management system

Each participating breast centre was to implement and maintain a quality management system (QMS) for which mandatory certification to ISO 9001 or a similar standard (KTQ) was to be obtained from a German third-party certifying body.

### DKG-commissioned certification service provider

An independent DKG-commissioned certification service provider (DKG-CSP) was to be created for the purpose of providing all necessary services and organisational tasks related to the certification of cancer services delivery, including consultancy, organising training courses for the specialist auditors and the conduct of audits.

### Certification procedure

The details of a procedure were to be work out which would result in dual certification of applicant breast centres to the DKG/DGS Requirements (FAB) as regards breast services delivery and to ISO 9001 (or similar, e.g. KTQ) as regards the mandatory QMS.

### Data collection and analysis

Data collection was based on voluntary registration of breast cancer treatment institutions with the DKG/DGS certification programme.

Data analysis was performed using standard software, including Microsoft Access^®^, Excel^® ^and Word^® ^2002 and 2003 (Microsoft Corporation, Redmond, WA, USA).

The relevant study data (including date of application for certification, date of initial certification, date of full certification and date of recertification) for each participating breast centre or hospital breast care unit were collected by the DKG-commissioned certification service provider (DKG-CSP) on a monthly basis.

Changes in the number of participating centres during the period from 1 December 2002 to 30 June 2008 were analysed by descriptive numerical and graphical methods (tables and graphs) and visual inspection. No statistical tests were employed.

## Results

### Phase I

**FAB**: Starting from a "checklist" catalogue of requirements in 2000, the German Cancer Society (DKG) and German Society of Senology (DGS) developed a comprehensive set of service delivery-related Requirements of Breast Centres (FAB) in several steps in collaboration with other relevant German specialist medical societies, including the German Society of Obstetrics and Gynaecology (DGGG). In April 2003, the FAB received their final form as a questionnaire to be completed by breast centres as the initial step in the certification process. A revised FAB questionnaire was made available online in August 2006. Table 1 presents in condensed form the overall structure of the 2006 version of the questionnaire and its essential items. This version of the questionnaire encompassed 185 single items. Of these, 84 were quantifiable items, with 49 representing quantitative minimum requirements.

**Table 1:** The DKG/DGS Requirements of Breast Centres (FAB): structure and general overview

1	General information on the breast centre
	Structure of the network; tumour board/treatment planning; collaboration with doctors in private practice; access to support groups; psychosocial and psycho-oncological care; aftercare and follow-up; patient involvement; scientific research activities
**2**	**Information on radiology services**
	Mammography equipment; stereotactic biopsy requirements; magnetic resonance imaging; breast ultrasound; radiography assistants; specialist radiologists (min. 2 names); basic and continuing medical education; quality circles (≥ 4 minuted meetings per year); number of mammograms read (> 2000/year (per breast)); specimen radiography; percutaneous biopsies (number); image-guided localisations (number); ductography (galactography) (images/year); description of techniques and procedures used; guidelines (fulfilment of requirements)

**3**	**Information on nuclear medicine services**
	Medical laboratory assistant (min. 2 names); specialist doctors (min. 2 names); continuing education for medical and paramedical staff; quality circles (≥ 4 minuted meetings per year); number of bone scintigrams (1st/3rd year requirement: > 200/> 400); sentinel node biopsies (SNBs, (1st/3rd year requirement: > 20/> 30); SNB detection rate (1st/3rd year requirement: > 80%/> 90% (gamma probe guided)), (1st/3rd year requirement: > 80%/> 90% (scintigraphy; optional)); quality control testing of equipment; fulfilment of relevant level-3 guideline requirements

**4**	**Information on surgical treatment – surgery – gynaecology – specialist breast services**
	Inpatient care; description; sufficient time for patients to consider treatment choices between core biopsy results and surgery (max. 14 days); operating theatre (OT) for breast surgery (min. 1 OT); continuing education of nursing staff; nursing staff (min. 2 full-time nurses/100 primary cases); specialist cancer nurse (min. 1); basic and continuing education for medical and paramedical staff; specialist doctors for the breast centre (min. 2 names); breast surgeons (min. 2 with specialist qualifications); details of breast surgeons' qualifications; quality circles (≥ 4 minuted meetings per year); number of primary breast cancers per surgeon: > 50 per year; total number of surgical procedures (axillary dissections (1st/3rd year requirement: > 85%/> 95%), revision procedures (< 5%), postoperative wound infections (2.5-max. 5%)); number of operations for breast tumours (benign, precancerous, primary, recurrences), primary carcinomas per centre per year (1st/3rd year requirement: > 100/> 150); number of pTis (1st/3rd year requirements: > 10%/> 15%); number of benign/malignant open biopsy findings; postoperative specimen radiography of microcalcifications after preoperative marker placement > 95%; rate of breast-conserving surgery (1st/3rd year requirement for pT1: > 50%/> 70%); mastectomy rate (1st/3rd year requirement for pT1: < 50%/< 30%); primary surgical treatment involving 1, 2, 3 or > 3 procedures, and rate of R1 resections; mean number of removed lymph nodes > 10 (in accordance with the guideline); breast reconstruction (responsibilities, details of collaboration if performed elsewhere, type of reconstruction procedure, surgeon's qualifications, general reconstructive surgery requirements); patient information and discussion of treatment options; breast clinics at least once weekly for early detection, treatment planning, advice to outpatients considering reconstruction, advice on benign breast disease, inflammation and impaired development (waiting times for clinic appointments/consultation < 2 weeks/1 hour); biopsies for histology (results after < 6 days); histological confirmation of tumour status (by core biopsy) in 90% of palpable and 70% of nonpalpable tumours; communication of tumour status diagnosis within < 1 week; documentation of the number of patient who refuse treatment; side-effects of treatment; knowledge and implementation of level-3 guideline.

**5**	**Information on radiotherapy services**
	High energy radiotherapy equipment (minimum specifications, other requirements); description of radiotherapy techniques (guideline-concordant dose regimen); radiography assistants (min. 2); continuing education for medical and paramedical staff; quality circles (≥ 4 minuted meetings per year); specialist radio-oncologists (min. 2); aftercare and follow-up; documentation/tumour assessments, reactions to radiotherapy (acute, subacute, late); compliance with level-3 guideline for treatment; written patient information during and after radiotherapy; applicable level-3 radiotherapy guidelines

**6**	**Information on pathology services**
	Specialist pathologists (min. 2 names); qualifications: details of expertise in breast histology and cytology; continuing education for medical and paramedical staff; external quality assurance; quality circles (≥ 4 minuted meetings per year); specialist experience: examination of 200 routine histological specimens from breast disease patients and 3000 histological specimens; rapid frozen sectioning (infrastructure, cryostat); number of rapid frozen sections performed per year; time to result; lymph node examination; specimen storage time: paraffin blocks ≥ 10 years, wet specimens ≥ 4; weeks; gross, microscopic and immunohistochemical examination and diagnosis; standardised processing for gross examination according to level-3 guideline; pathologist's report on breast specimens (except diagnostic core biopsies) must contain guideline-specified details for the gross pathology report microscopic examination; resection/safety margins; pT and pN status for > 95% of invasive tumours; measurable receptors (hormone receptors (> 95%), HER2/neu (> 95%), FISH analysis if necessary)

**7**	**Information on oncology services (gynaecology, medical oncology, inpatient/outpatient services)**
	Specialist oncologist (internist or gynaecologist, experienced in chemotherapy (≥ 800 treatment cycles) and endocrine, immunological, adjuvant, palliative and supportive therapy and treatment of side effects); quality circles (≥ 4 minuted meetings per year); continuing education for medical and paramedical staff; ≥ 50 breast cancer chemotherapies/year per treatment unit or partner, or ≥ 200 chemotherapies/year for various cancers; provision of inpatient and outpatient chemotherapy; appropriate infrastructure; min. 2 chemotherapy rooms; description of facilities for supportive/palliative care; description of treatment phases during chemotherapy (initiation to termination); provision of information to patients and dialogue with patients; compliance with relevant level-3 guideline requirements

**8**	**Tumour documentation/outcome quality**
	Details of tumour documentation system (TDS), which must contain complete patient and treatment details for ≥ 3 months prior to initial certification, details of treatment stage, data for cancer registries; guideline-compliant data sets; data collection by calendar year and certification period; responsible documentation manager; 50% position/breast centre for data collection-related tasks; data selection options must include by year, patient's name, diagnosis, type of treatment, date of recurrence/metastasis, survival data; outcome quality indicators: disease-free survival (DFS), overall survival (OAS), date and proportion of recurrence per stage and type of surgery (breast-conserving surgery (BCS) vs. mastectomy); date and location of metastasis; quality of life; Kaplan-Meier curves (local recurrence-free survival and OAS, by relevant prognostic groups, survival from progression); comparisons with other breast centres; multivariate analyses; appraisal of achievement of TDS objectives (transparency); DFS and OAS must be available at recertification every 3 years; 10-year recurrence rates for mastectomy/BCS: < 10%/< 15%; completed questionnaire and relevant process descriptions must be available at initial certification; documented data must be accessible; uses for TDS data: at least once-yearly in-house analysis of the data, centre-specific and comparative analyses, analysis-based improvements; archiving of results (data analysis, appraisal, actions); discussion of results with the main collaborating partners and the breast centre network as a whole; compliance of data with guideline requirements; responsible physicians' awareness of their data compared with other centres and the literature (quality of data, quality of care); appraisal of flexibility of documentation

The FAB of 2006 encompass 185 items from eight main areas. Applicant breast centres need to (1) describe existing facilities, resources and procedures and (2) meet specified requirements in order to attain initial or full certification and recertification.

In brief, the DKG/DGS Requirements of Breast Centres (FAB) [13] comprise a catalogue of cancer services requirements, i.e. clinical practice and service implementation criteria, which cover the following aspects:

• general criteria (multidisciplinarity, access to support groups, psychosocial care),

• staffing (criteria relating to different disciplines such as radiology, nuclear medicine, surgical disciplines, radiotherapy, pathology, oncology),

• national level-3, evidence-based guidelines for service delivery, and

• documentation, data analysis and outcome.

The FAB are reviewed, revised and developed further on a regular basis by the DKG-appointed Certification Committee, whose main task is to ensure that the FAB reflect current guidelines and other, e.g. legal, requirements.

**QMS**: All participating breast centres maintained a QMS that was certified to an internationally recognised standard, usually ISO 9001 (71%), or the German KTQ standard for health care providers http://www.ktq.de.

**DKG-CSP**: During an initial phase, audits were conducted by DKG/DGS-appointed specialists, senior doctors from hospitals other than the applicant breast centre, and certification was granted by the DKG. In 2003, the DKG founded a separate certification service provider, OnkoZert GmbH, Ulm, Germany, (referred to here as the DKG-CSP) to manage the entire certification process, i.e. to handle the applications, organise courses to train Specialist Expert auditors, schedule the audits and issue the certificates on behalf of the DKG and the relevant specialist medical societies, e.g. the DGS for breast centres. The DKG-CSP became fully operational as a separate service provider in December 2004.

The DKG-CSP operates under the supervision of the DKG and the relevant specialist medical societies and acts on the decisions of the Certification Board.

**Figure 1:**
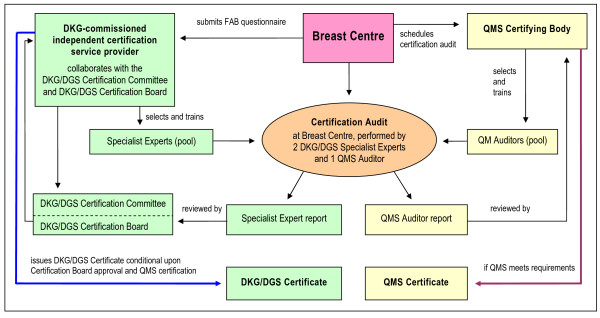
**Dual certification of breast centres to the DKG/DGS Requirements (FAB) and an accepted QMS standard.** Adapted from [23] and [11]. DKG = German Cancer Society; DGS = German Society of Senology; QM(S) = quality management (system).

**Certification procedure**: During the initial phase from November 2001 to June 2003, test and pilot certifications were performed. After the inception of the DKG-CSP, the current procedure was implemented in July 2003. A schematic representation of the dual certification procedure for breast centres is shown in Figure 1. The procedure begins with the applicant breast centre submitting to the DKG-CSP an enquiry form requesting initiation of the certification process. The DKG-CSP will then estimate the effort and cost of certification, upon which the breast centre will file a formal application and submit the completed FAB questionnaire. This information is assessed for FAB compliance by DKG-CSP-trained Specialist Experts, who will subsequently endorse the application or suggest necessary changes in preparation of the certification audit. The Specialist Experts will then arrange an on-site preliminary meeting at the applicant breast centre to discuss unclear items or critical issues that might endanger certification. Certification is then scheduled according to an audit plan. At the actual, usually two-day certification audit, two Specialist Experts and one expert from the QMS certifying body will inspect the various departments, facilities and areas of the applicant breast centre that are relevant to multidisciplinarity, and any external partners with which it collaborates. These experts will also inspect randomly sampled documents and records and interview staff to verify the information submitted in the FAB questionnaire. In the event of discrepancies, the breast centre is given up to three months to rectify these prior to re-audit. The on-site certification audit is concluded with a final discussion, at the end of which the experts inform the breast centre whether they will recommend that the DKG/DGS Certification Board endorse certification and whether the QMS requirements have been met. Once the Certification Board has endorsed the application and QMS certification has been obtained, the DKG Certification Board will issue the DKG/DGS Certificate. To monitor FAB compliance, annual on-site audits based on up-dated FAB questionnaire data are conducted randomly by Specialist Experts, with a particular focus on comments, suggestions and issues from previous audits. The various audits and re-audits are conducted by different Specialist Experts.

The DKG/DGS Certificate, including the QMS certificate, is valid for three years. The breast centre then needs to apply for recertification, which involves an FAB-based audit similar to the initial procedure, and QMS certification to the ISO 9001 or the KTQ standard [11,23].

In summary, DKG/DGS certification of breast centres is a two-part procedure involving certification of the mandatory hospital QMS to the ISO 9001 or the KTQ standard, and certification of service delivery to the DKG/DGS Requirements of Breast Centres, the FAB. The DKG/DGS Certificate expires after three years, necessitating recertification according to the same basic procedure.

### Phase II

**Proof of concept:** The first pilot certification of a breast centre to both ISO 9001 and DKG/DGS Requirements, then still the "checklist" in its 4th revision, was achieved in December 2002. The dual certification procedure in its present form was established in July 2003 after the 5th revision of the "checklist", which was then renamed to FAB.

**Figure 2:**
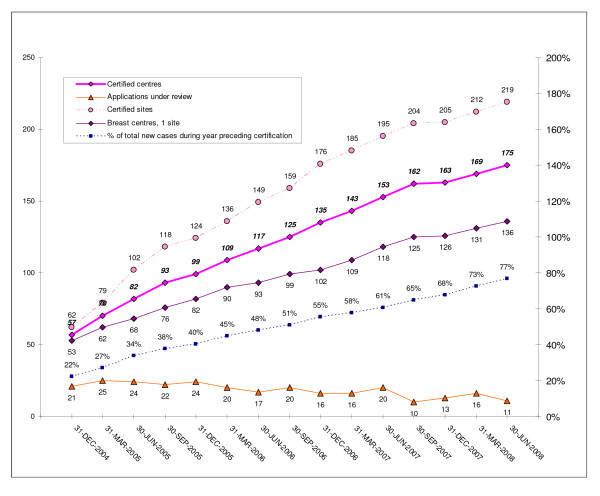
**DKG/DGS-certified breast centres: applications, certified centres and sites, and primary cases/year treated at certified centres**.

Figure 2 summarises the certification-related data collected by the DKG-CSP since it became fully operational in November 2004.

### Phase III

Final proof of concept was provided by the first recertifications. At the end of 2005, the first two DKG/DGS-certified breast centres successfully completed recertification after their first three-year certification. By mid 2008, 79 of 80 breast centres due for recertification had successfully completed the process. One breast centre failed to meet the recertification requirements in 2007 and was therefore excluded from further participation in the DKG/DGS certification programme.

### Phase IV

**Further results:** From the end of 2004 to 30 June 2008, the overall number of certified breast centres increased from 57 to 175, while the number of single-site certified breast centres rose from 53 to 136 (cf. Figure 2). In relative terms, the proportion of single-site breast centres dropped from 93% (53/57) to 78% (136/175) from the end of 2004 to mid 2008. This was due to rising numbers of 2- to 4-site centres joining the DKG/DGS dual certification programme. During the same period the number of certified breast centres with 2, 3 or 4 sites increased from 4 to 39, the vast majority of which (36/39; 92%) were 2-site centres.

During the 3.5-year period from 1 January 2005 to 30 June 2008, the mean number of primary cases per breast centre in the year preceding certification rose by 6% from 196 to 208, while the total number of primary breast cancers treated at breast centres with dual ISO 9001 (or KTQ) and DKG/DGS certification in Germany during the year preceding certification increased 3.5-fold from 11,152 to 38,485 (data not shown), representing a rise from 22 to 77% (see Figure 2) of an estimated 50,000 primary breast cancers per year.

## Discussion

Breast cancer continues to be the most common cancer in women, both in Germany and throughout the world [24,25]. With more than 50,000 women estimated to be diagnosed annually with breast cancer, Germany ranked ninth in a 2002 comparison of 24 European countries [25].

In recent years, and in line with European policies [6,7], health policies in Germany have emphasised the increasing importance attributed to breast cancer [26,27]. Efforts have been directed towards developing and implementing structured, intersectoral quality management programmes aimed at optimising breast cancer care to reduce inappropriate care and the over- and underprovision of care [28].

Until the initiation of the present study in 2002, the term "breast centre" was not in any way properly defined or protected in Germany. Therefore the German Cancer Society (DKG) and the German Society of Senology (DGS), in collaboration with other relevant specialist medical societies, jointly undertook to develop and implement a definitive certification programme for multidisciplinary breast centres [12] with a view to establishing a prototypical certification programme for other multidisciplinary site-specific cancer centres such as colon and prostate centres [29,30].

*Phase I *of the present study was successful in developing a set of specific Requirements of Breast Centres (*Fachliche Anforderungen an Brustzentren*; FAB) which were largely based on the two national level-3, evidence-based guidelines on early detection [16] and the diagnosis and treatment of breast cancer [15,14], but were also compatible with the EUSOMA Requirements of a Specialist Breast Unit [8,9]. Compared with the EUSOMA Requirements, however, the FAB are more comprehensive in terms of the number of individual requirements a breast centre must meet. The number was even increased in 2006 from 173 items to 185 single items (including 84 quantifiable requirements and 49 quantifiable minimum requirements) as compared with 67 EUSOMA items (including 10 quantifiable minimum requirements). This difference in detail and comprehensiveness is likely due to the EUSOMA requirements representing an international effort that needs to take account of, and bridge, the differences between national healthcare systems. The differences between the DKG/DGS and EUSOMA requirements have been discussed in detail elsewhere [11]. Generally speaking, the German FAB can be considered a further development and expansion of the EUSOMA Requirements.

This first phase of the study was also successful in that all participating centres subjected their legally required quality management system (QMS) to third-party certification with annual audits. In this respect the DKG/DGS certification programme differs from EUSOMA certification, which does not require that breast centres have a QMS.

Finally, study phase I was successfully concluded with the founding of a certification service provider by the German Cancer Society (DKG) to manage the organisation and conduct of the certification process, and with the development of the dual certification procedure involving QMS certification to an internationally accepted standard such as ISO 9001 in addition to certification to the FAB requirements developed by the DKG/DGS. The requirement of a QMS as such, and even more so, a third-party-certified QMS constitutes a significant difference between the DKG/DGS and the EUSOMA certification programmes. Moreover, DKG/DGS certification involves closer, annual auditing and recertification at 3-year intervals as opposed to the EUSOMA system with 5-year recertification.

As of mid 2008, 22 German breast centres and two Swiss breast centres had been granted EUSOMA Initial Certification but none had attained EUSOMA Full Certification requiring five years of audit data, or Re-Certification according to the procedure outlined in the revised version http://www.eusoma.org/doc/EusomaCertificationDocument.pdf of the relevant 2006 EUSOMA position paper [31]. By contrast, as of 30 June 2008 the number of breast centres with dual certification to ISO 9001 and the DKG/DGS FAB was 175, indicating that the programme has rapidly gained wide acceptance since its inception in 2002.

*Phase II *of the study, i.e. the pilot certification of a small number of breast centres to the FAB in conjunction with separate mandatory third-party certification of a previously implemented QMS, was also completed successfully.

*Phase III *similarly demonstrated successful proof of concept for the DKG/DGS certification programme by completing recertification of the pilot breast centres.

*Phase IV *finally extended the proof of concept to the multicentre level by achieving certification and recertification of numerous other breast centres in Germany, the total number of DKG/DGS-certified breast centres reaching 175 by mid 2008.

The results of the present study thus demonstrate the feasibility and general acceptance of the voluntary dual certification programme for breast centres initiated and implemented under the auspices of the DKG and the DGS as one major strategy to improve quality assurance in breast cancer services in Germany.

The cornerstones of the current approach to quality assurance in cancer services advocated by the DKG, the DGS and other relevant specialist medical societies in Germany today are multidisciplinarity, specialisation, centralisation and benchmarking, with voluntary certification representing the DKG's official "seal of quality".

*Multidisciplinarity*, in the prototypical case of breast cancer, is based on the rationale to give all women with this disease access to all areas of expertise necessary to offer them the best possible care throughout all phases of their treatment.

*Specialisation *in the broader sense involves the provision of multimodal and individualised diagnostic and therapeutic strategies. In the narrower sense specialisation is defined as a surgeon's caseload within a fixed time period. A significant body of evidence supports the conclusion that the overall survival rate increases with multidisciplinary care and the level of surgeon specialisation, leading to declines in both mortality and morbidity [18,19,32-37]. In addition to hospital volume, another important parameter is annual surgical caseload. Sainsbury et al. showed that 5-year survival from breast cancer was significantly better for surgical caseloads > 30 cases/year in conjunction with a full range of treatment options [32]. Similarly, Stefoski Mikeljevic and associates more recently found that patients treated by low-workload surgeons had poorer 5-year survival than those treated by surgeons with annual workloads of > 50 new patients [38]. Professional organisations have also included recommendations for minimum caseloads of breast cancer patients in their guidelines. The European Society of Breast Cancer Specialists (EUSOMA), for instance, requires that surgeons at specialist breast units personally perform primary surgery on at least 50 newly diagnosed breast cancers per year but does not base this figure on any published evidence [8,9]. Also, it is gradually emerging from the literature that multidisciplinarity – but only in the context of breast cancer – appears to be more important than surgeon's specialisation.

*Centralisation *of breast cancer treatment in hospitals that treat a minimum number of breast cancer cases per year can also significantly improve survival rates. In a study of the 5-year survival rates of breast cancer patients from 266 hospitals in New York State, Roohan and associates [18] found that, compared with "high volume hospitals" performing 150 surgeries/year, the mortality risk associated with treatment in "very low volume hospitals" (< 10 surgeries/year) was 60% higher, whereas in "low volume hospitals" (11–50 surgeries/year) and "moderate volume hospitals" (51–150 surgeries/year) the mortality dropped to 30% and 19%, respectively. In this context it should be critically remarked that the study by Roohan et al., to our knowledge, is the only source in the pertinent literature to provide evidence-based support for a minimum annual hospital volume of 150 primary breast cancers. More recently a Canadian study by Hébert-Croteau and collaborators [39] confirmed for 7-year survival from breast cancer that annual hospital volumes of 100 new cases were associated with better survival rates than lower hospital volumes. In Austria, a negative effect on survival rate was found in patients treated at small departments [40]. Moreover, patients treated at a hospital located less than 10 miles from their homes were found to have a 10% higher mortality risk and those who travelled more than 10 miles were more likely to travel to high-volume hospitals [18]. Centralisation also offers better opportunities for clinical studies, which have been shown to improve outcomes, e.g. in Austria [41]. Further evidence of the benefits of centralisation has been found in other countries, e.g. in England [36].

These findings prompted a major reassessment of the organisation of the provision of treatment. The internationally accepted goal now is centralisation or, failing this, the establishment of tightly co-ordinated networks of cancer services providers capable of multidisciplinary co-operation with a high degree of specialisation and maximum quality assurance. The European Parliament sees specialised breast centres as a crucial factor in the effort to reduce the mortality rate by up to 25% and therefore called for the necessary basis to be created for breast centres to be introduced by 2008 [6,7]. Multidisciplinarity is reportedly associated with an 18% reduction in mortality provided that all specialists cooperate optimally [35]. This is due to the fact that surgical treatment of breast cancer, at least when it comes to mastectomy, is less complex and sophisticated than surgery for ovarian cancer or colon cancer. In the latter case, for example, recent literature analyses have shown short-term outcome and long-term survival to depend on surgical and hospital caseload [42,43]. Hence the hypothesis emerges in all discussions on the subject that it is surgical caseload that matters in colon and ovarian cancer whereas in breast cancer the crucial factor is the caseload of the whole specialist team, including the radiologist, pathologist, surgeon, oncologist and radio-oncologist.

Costs and economic viability, however, are another fundamental factor to be considered when demanding centralisation of breast cancer care in high-volume specialist breast centres. Centralisation implies that a sufficiently high caseload can be generated to enable a "centre" to provide adequate structural and process quality in terms of both infrastructure and financial means to allow all relevant specialists involved in the diagnosis and treatment of breast cancer to work together under one roof. To what extent the initially high costs of this new evidence-based standard and the associated quality management systems will be feasible Europe-wide and in what time frame, still remains to be clarified. An economic analysis by Pagano et al. [44] estimated that to justify the economic investment, multidisciplinary breast cancer centres must achieve a minimum annual volume of 200 newly diagnosed cases of breast cancer. Similarly, EUSOMA also calls for an annual caseload of at least 150 newly diagnosed cases of primary breast cancer in order to ensure cost effectiveness and maintain the expertise of specialised multidisciplinary teams [8,9]. The greatest cost factor would appear to be that associated with multidisciplinarity, in particular the availability, at short notice, of all necessary disciplines. These estimates and requirements are in stark contrast with calculations and considerations published by Beckmann and associates [45,46,29,30] who argue that breast cancer centres in Germany are not economically viable without substantial cross-subsidisation from other health care services, e.g. night duties and obstetric services provided by gynaecologists. In the medium term, cost-benefit analyses and outcome data will be needed to decide on the economic viability of breast centres.

*Benchmarking *the quality of breast cancer care, lastly, is the final cornerstone in current voluntary endeavours in Germany to implement quality assurance in breast cancer services and thus to optimise the quality of diagnosis and treatment. Therefore the DKG/DGS certification programme itself also requires the collection of structure-, process- and treatment-related data. Each participating breast centre is assessed on the basis of these data and has to demonstrate improvements at the yearly interim audits and the three-yearly recertification audits. The progress achieved within a voluntary programme for benchmarking the quality of breast cancer care in Germany, which was developed and implemented in parallel with the present certification programme, has been reported elsewhere [17].

*The potential limitations of the present study *could include the following. Fundamental criticism levelled at the centralisation of breast cancer treatment at large cancer hospitals and the subsequent certification of these breast centres concerns the fact that no definitive measures exist as yet to demonstrate the advantages of this system. Furthermore, the differences between the various treatment modalities at the different large centres are likely to be small, possibly even statistically nonsignificant. Therefore it may never be possible to obtain conclusive evidence of differences in long-time survival and recurrence rates. It may well be that EUSOMA and all the certification specialists will never be able to prove their case. Outcome quality would need to be measurable using these endpoints, just as structural and process quality can be assessed via quality management manuals. However, it is unlikely that comparisons at the level of individual breast cancer centres will be possible before 10-year data become available. Hence such comparisons of centres may yet prove very difficult, if not impossible. As demonstrated with regard to benchmarking the quality of breast cancer care [17], outcome quality can so far only be measured indirectly by using surrogate parameters under the general assumption that better short- to medium-term structural quality and process quality will result in improved long-term outcome quality. In other words, the currently used surrogate parameters are also, or simply, measures of guideline compliance that the specialist teams introduce to the individual centres on the basis of structural and process quality, and multidisciplinarity.

As regards guideline compliance, DKG/DGS certification, unlike EUSOMA certification, involves an accurate assessment of guideline compliance by independent Specialist Experts on the basis of each centre's individual quality management manual. Such audits by Specialist Experts can subsequently be certified by impartial, third-party ISO certification experts. Essentially, the audits of guideline compliance are underpinned by available surrogate parameters for benchmarking.

The maintenance and further development of a quality management system (QMS) at a breast centre initially requires substantial investments in terms of funds, staff and extra administrative time. Moreover, the benefit of these quality assurance measures does not become evident to a team until they have been in place for some time and the quality management tasks become an integral part of the hospital routine. This large initial investment, however, is offset by the team's growing skill in implementing the QMS and the systematic optimisation of the treatment procedures, which eventually become evident to all, not least due to increasing numbers of patients. This system enables a centre to present its statistics and efficiency to the public and compete with other centres.

According to the DKG/DGS certification procedure, smaller centres can undergo initial, first-year certification with approx. 100 new cases of primary breast cancer. However, in addition to the general increase in standards these centres are expected to meet in order to achieve full certification after three years, they must also increase the annual number of new cases of primary breast cancer to at least 150.

### Future prospects

The German Cancer Society (DKG) recently presented an overall plan for the long-term development of cancer services in Germany [47]. Following the prototypical example of the breast centres, further site-specific multidisciplinary cancer centres are currently being set up and applying for voluntary DKG certification. These include centres for colon, prostate, lung, skin and gynaecological cancers. The first colon, prostate, gynaecological and skin cancer centres received DKG certification in March 2006, November 2007, May 2008 and January 2009, respectively.

These site-specific cancer centres form the lowest rung of a three-tier service structure. The second level will consist of larger, intermediate-level cancer centres comprising several site-specific cancer centres. The third and highest level in this model for the provision of quality-assured cancer services is that of the "comprehensive cancer centres" (CCCs). These will be responsible for research, forging close links between preclinical research and clinical oncology. Based on the American model, the CCCs combine the tasks of both the site-specific cancer centres and the general cancer centres.

## Conclusion

Nationwide voluntary certification of breast centres is feasible and well accepted in Germany. Dual certification of breast centres that involves certification of breast services to a set of guideline-derived requirements (FAB) in conjunction with separate, voluntary, third-party certification of a legally required quality management system can serve as a model for other multidisciplinary site-specific cancer centres.

The DKG/DGS certification procedure for multidisciplinary breast centres in Germany has, in fact, already become the model scheme for quality assurance and centralisation in the broader field of oncology. While the goal is now well advanced of establishing a nationwide network of certified breast centres, the important next step for the centres will be to achieve recertification, since this demands even higher standards. Beyond that, certified breast centres play an important role within broader collaborative networks which include gynaecologists working in private practice, mammography screening centres and health professionals in associated disciplines.

The promotion of quality in cancer services at both the national and European level will remain an important task for multidisciplinary and specialist medical societies in the immediate future.

## Abbreviations

**AGO**: Arbeitsgemeinschaft Gynäkologische Onkologie (DGGG Working Group on Gynaecological Oncology); **BCS**: breast-conserving surgery; **BCT**: breast-conserving therapy; **DGGG**: Deutsche Gesellschaft für Gynäkologie und Geburtshilfe (German Society of Obstetrics and Gynaecology); **DFS**: disease-free survival; **DGS**: Deutsche Gesellschaft für Senologie (German Society of Senology); **DKG**: Deutsche Krebsgesellschaft (German Cancer Society); **DKG-CSP**: DKG-commissioned certification service provider; **DOC**: Deutsches Onkologie Centrum Holding GmbH (German Oncology Centre Ltd.); **EUSOMA**: European Society of Breast Cancer Specialists (formerly: of Mastology); **FAB**: Fachliche Anforderungen an Brustzentren (Requirements of Breast Centres); **FISH**: fluorescent in situ hybridisation; **HER2/neu**: human epidermal growth factor receptor-2; **ISO**: International Organization for Standardization; **KTQ**: Kooperation für Transparenz und Qualität im Gesundheitswesen (Cooperation for transparency and quality in health care); **OAS**: overall survival; **OT**: operating theatre; **pN**: pathologically determined nodal status; **pT**: pathologically determined tumour stage; **pTis**: carcinoma in situ (pathologically staged); **QA**: quality assurance; **QI**: quality indicator; **QMS**: quality management system; **R1**: microscopically positive surgical margin; **SNB**: sentinel node biopsy.

## Competing interests

The authors declare that they have no competing interests.

## Authors' contributions

SYB participated in the development of the reported certification programme, compiled and analysed the reported data, and drafted the manuscript. SYB and MB contributed equally and should be considered joint authors. MB, WJ, MWB and RK participated in the development of the reported certification programme and reviewed the draft manuscript. AK participated in data analysis and reviewed the draft manuscript. RK was also responsible for the development of the Requirements of Breast Centres (FAB) on behalf of the German Cancer Society (DKG) and the German Society of Senology, and reviewed the draft manuscript. DW participated in the development of the reported certification programme, conceived of the present study and its design, outlined the contents of the manuscript and reviewed the draft manuscript. RK and DW contributed equally and should be considered joint authors. All authors read and approved the final manuscript.

## Pre-publication history

The pre-publication history for this paper can be accessed here:

http://www.biomedcentral.com/1471-2407/9/228/prepub
